# A new signature based on alternative polyadenylation for prognostic prediction and therapeutic responses in low-grade glioma

**DOI:** 10.18632/aging.203844

**Published:** 2022-01-18

**Authors:** Yuzhi Wang, Yunfei Xu, Yi Zhang

**Affiliations:** 1 Department of Laboratory Medicine, People’s Hospital of Deyang City, Deyang 618000, Sichuan, China; 2 Department of Laboratory Medicine, Chengdu Women’s and Children’s Central Hospital, Chengdu 610031, Sichuan, China; 3 Department of Blood Transfusion, People’s Hospital of Deyang City, Deyang 618000, Sichuan, China

**Keywords:** low-grade glioma, alternative polyadenylation, prognosis signature, therapeutic responses, TCGA

## Abstract

Background: Evidence from research supports the significant role of alternative polyadenylation (APA) in the development of cancer. The aim of this study is to explore the prognostic and therapeutic value of APA events for patients with low-grade gliomas (LGG).

Methods: The gene expression and APA profiles of patients with low-grade gliomas were obtained from The Cancer Genome Atlas database. All patients were sorted randomly into training and test sets. The prognostic-associated events of alternative splicing were screened by univariate Cox regression. Subsequently, Least Absolute Shrinkage and Selection Operator and multivariate Cox analysis were performed to construct a prognostic signature. The patients were sorted into the high and low-risk groups based on their median risk score. Bioinformatics methods were used to identify genetic variation, pathway activation, immune heterogeneity, and drug response differences between the two groups.

Results: A prognostic signature was constructed shown to be capable of accurately predicting prognosis of patients with LGG. Notable variations were observed in the tumor mutation burden and copy number variations between the high-risk and low-risk patients. Besides, the high-risk group had enhanced immune cell abundance and immune checkpoint gene expression. In terms of drug response, we further found that the patients of high-risk group were more sensitive to immunotherapy, but chemotherapy was suggestively more appropriate for the low-risk group patients.

Conclusion: Our findings give new insights and methods related to prognosis prediction and treatment methods for LGG patients, and expand the understanding regarding the role of alternative splicing in LGG.

## INTRODUCTION

Glioma is the most common primary intracranial tumor in the adult central nervous system with high recurrence and mortality rates [1]. The age-calibrated incidence of gliomas ranges from 4.67‱ to 5.73‱ [2]. As per the classification system of the World Health Organization (WHO), gliomas can be sorted into grades I–IV, of which grades II and III are defined as low-grade gliomas (LGG) [3]. Although LGG is less malignant as compared to GBM, seemingly the recurrence of the tumor and malignant progression cannot be evaded completely even after standard treatment such as surgical resection, radiotherapy, and chemotherapy is received by the patient [3]. Because of tumor heterogeneity, the prognosis of LGG patients varies widely, with survival periods ranging from 1 to 15 years [4]. As a result of the heterogeneity of LGG patients, there is a need for the development of effective biomarkers that can be used to stratify predictions based on patient prognostic risk, thereby facilitating the development of precise treatment regimens.

The alternative polyadenylation (APA) is a very common RNA processing mechanism that occurs during pre-mRNA maturation. Multiple mature mRNA subtypes can be generated from the same pre-mRNA by selecting different polyadenylation signaling sites (PAS) in the 3′-UTR [5]. Whole-genome deep sequencing indicates that at least 70% of multiple transcripts coded by human genes are derived as a result of APA [6]. The APA plays a significant function in controlling the stability of mRNA, its localization and translation, protein-coding, and the localization of protein [7]. According to recent studies, the change of APA is closely associated with the occurrence and development of different tumor types, for instance, low expression levels of PCF11 in neuroblastoma is related to extensive APA in the transcriptome, good prognosis of the patient, and spontaneous tumor regression, while PCF11 knockdown causes abnormal neural differentiation [8]. During glioblastoma, the down-regulation of CFIm25 results in 3′UTR shortening and the up-regulation of oncogenes such as CCND1 and Pak1 which increases tumorigenicity and enhances tumor size [9, 10]. Though the biological significance of APA in tumors is widely accepted, its prognostic value and biological function in LGG are still largely unknown.

In this research, a systematic analysis of APA events in LGG patients was performed using The Cancer Genome Atlas (TCGA) database and we identified numerous overall survival (OS) related APA events. The potential biological functions of these OS-related APA events were explored in detail. Subsequently, a signature based on multiple prognostic APA events was constructed to predict the prognosis of LGG patients. Furthermore, we analyzed the correlation between the signature and tumor immunity, tumor mutation burden (TMB), and copy number variations (CNVs). The value of this signature in predicting the response of LGG patients to various treatments was also evaluated. At the end of this study, a core regulator (CR)-APA network was constructed to disclose the underlying mechanism through which the events of APA affect LGG prognosis. The findings of this research may help in understanding mechanisms involved in the occurrence and development of LGG.

## MATERIALS AND METHODS

### Data collection and preprocessing

The expression profiles, mutations, CNVs, and clinical information of LGG patients were downloaded from the TCGA database. Moreover, data for APA events were obtained from the UCSC Xena database (https://xena.ucsc.edu). Patients were then randomly sorted into two sets namely a training set and a test set in the ratio of 1:1 by the software. The percentage of distal polyA site usage index (PDUI) value is an intuitive ratio from 0 to 1 that was used for the quantification of events related to APA. A set of strict filtering criteria (Percentage of samples with PDUI value ≥75%, mean PDUI value ≥0.05 and standard deviation PDUI value≥0.01) were established for ensuring the reliability of the subsequent analysis of APA events. Afterward, for the completion of missing data regarding APA, we used the k-nearest neighbor algorithm.

### Identification of prognostic APA events and functional enrichment analysis

For the determination of survival-related APA events, univariate Cox regression analysis was performed to evaluate the relation between APA events and the overall survival period of patients with LGG. The APA events with *P*-value <0.05 were chosen as survival-related APA events. The web tool Metascape (https://metascape.org/) was utilized for analyzing the functional enrichment of parental genes for survival-related APA events. Terms having a calibrated *P*-value <0.05 were taken as substantially enriched. Bar plots were used for visualizing key terms in the Kyoto Encyclopedia of Genes and Genomes (KEGG) pathways and Gene Ontology (GO) function enrichment analysis (including biological process (BP), cellular component (CC), and molecular function (MF).

### Construction of prognosis signature for patients with LGG based on APA events

At first, the least absolute shrinkage and selection operator (LASSO) analysis was performed on the top 20 most prognostic-related APA events to further screen key APA events and avoid overfitting of subsequent models. Afterward, a stepwise multivariate Cox regression analysis was carried out for the development of a prognostic signature for APA events. The risk value of each patient was measured as per the following formula: β1 × Exp1 + β2 × EXP2 + β I × EXPi, where β was the coefficient value and exp was the PDUI value of APA time. The risk value was an indicator that measured the prognostic risk of each LGG patient. All patients were sorted into two groups: the high-risk and low-risk groups according to their median risk values. The Kaplan-Meier survival analysis was carried out for the verification of survival differences among the high-risk and low-risk groups. The time-dependent receiver operating characteristic (ROC) curves were drawn for the evaluation of the predictive ability of APA signature.

### Somatic mutation and copy number analysis

This study used the “maftools” package (version 2.10) for calculating and visualizing the mutation data. TMB was identified as the total number of somatic non-synonymous mutations in the coding regions. For each sample, mutation detection was done using the preprocessed and annotated MAF data files generated by the varscan platform for the calculation of the tumor mutation burden. The GISTI is a tool that is widely used for the identification of genes targeted for somatic copy number changes that trigger the growth of cancer. In this research, the GISTIC 2.0 software was used to identify regions with significant gene amplification or deletion in the CNV data of LGG [11]. The parameter thresholds were defined as amplifying or missing length >0.1 and *P*-value <0.05.

### The evaluation of immune infiltration and immunotherapy response

Based on the expression profile information, the estimation of stromal and immune cells in malignant tumours using expression data (ESTIMATE) algorithm was used to generate matrix and immune scores to estimate the level of infiltrating matrix, immune cells, and tumor purity in LGG tissues [12]. In addition, the “GSVA” package (version 1.42) of Single-sample gene set enrichment analysis (ssGSEA) algorithm was used to quantify the enrichment scores of 16 immune cells and 13 immune functions for every sample of LGG. 14 genes have previously been reported as hub targets for immune checkpoint inhibitors [13]. Differences in the therapeutic efficacy of immune checkpoint inhibitors in malignancies are associated with differences in immune checkpoint gene expression. Therefore, the correlation between the expression levels of 14 immune checkpoint genes and our signature was analyzed in depth. Tumor immune dysfunction and exclusion (TIDE) is an algorithm that predicts response to immunotherapy based on mimicking tumor immune evasion mechanisms [14]. The algorithm was performed to preliminarily explore the possibility of each sample responding to immunotherapy.

### Clinical drug response prediction

Based on the Genomics of Drugs Sensitivity in Cancer (GDSC) cell line dataset, the R package “pRRophetic” (version 0.50) was used to predict the sensitivity of patients to signature-targeted chemotherapy agents. The R package can be evaluated by ridge regression for IC50 of included drugs, and the prediction accuracy can be observed by 10-fold cross-validation on the basis of the GDSC training set [15, 16].

### Establishment of the nomogram

The clinicopathological parameters of LGG patients were added in univariate and multivariate Cox regression analyses for the verification of the independence of the risk scores based on the survival-related APA signature. Afterward, a nomogram signature was constructed using all independent prognostic factors to develop a scoring system to evaluate the OS of patients at 1-year, 3-year, and 5-year. To demonstrate the effectiveness of the system, the C index curve, calibration curves, and time ROC curves were used for the evaluation of the recognition performance of the system. The DCA cures were used to assess the clinical applicability of the scoring system.

### Construction of correlation network between CR genes and APA events

A total of 22 CRs genes were obtained from previous studies [17] and CRs gene expression profiles were collected from the TCGA database. The Spearman correlation method was performed for the calculation of the correlation among the PDUI values and SF expression levels of survival-related APA events. *P*-value <0.001 and absolute values of correlation coefficient >0.5 were considered as thresholds. Lastly, to construct and draw the interaction network between APA and SF, the Cytoscape software was used in this study.

### Statistical analysis

The R software (version 4.0.5) was used for all statistical analyses, and *P*-value <0.05 on both sides was considered statistically significant. The Kaplan-Meier survival analysis was done by log-rank test. The independent sample *T*-test and Wilcoxon test were performed to compare the two groups. The language was polished by Bullet Edits Services (http://www.bulletedits.cn).

## RESULTS

### Overview of APA events in patients with LGG

The general analysis flow chart of this study was illustrated in Supplementary Figure 1. 511 LGG patients in total were enrolled in the current research, and their clinical data were compiled in Table 1. According to the above screening criteria, 6,574 APA events were identified from 6,214 genes, indicating that one gene can produce one or more APA events.

**Table 1:** Clinical characteristics of the TCGA cohort.

**Characteristics**	**Groups**	**Number (percentage)**
Age	<60	442 (86%)
	>60	69 (14%)
Gender	Male	283 (55%)
	Female	228 (45%)
Grade	G2	246 (48%)
	G3	264 (52%)
Histology	Astrocytoma	193 (38%)
	Oligodendroglioma	187 (36%)
	Mixed glioma	131 (26%)

### Survival-related APA events and the analysis of functional enrichment

**Figure 1:**
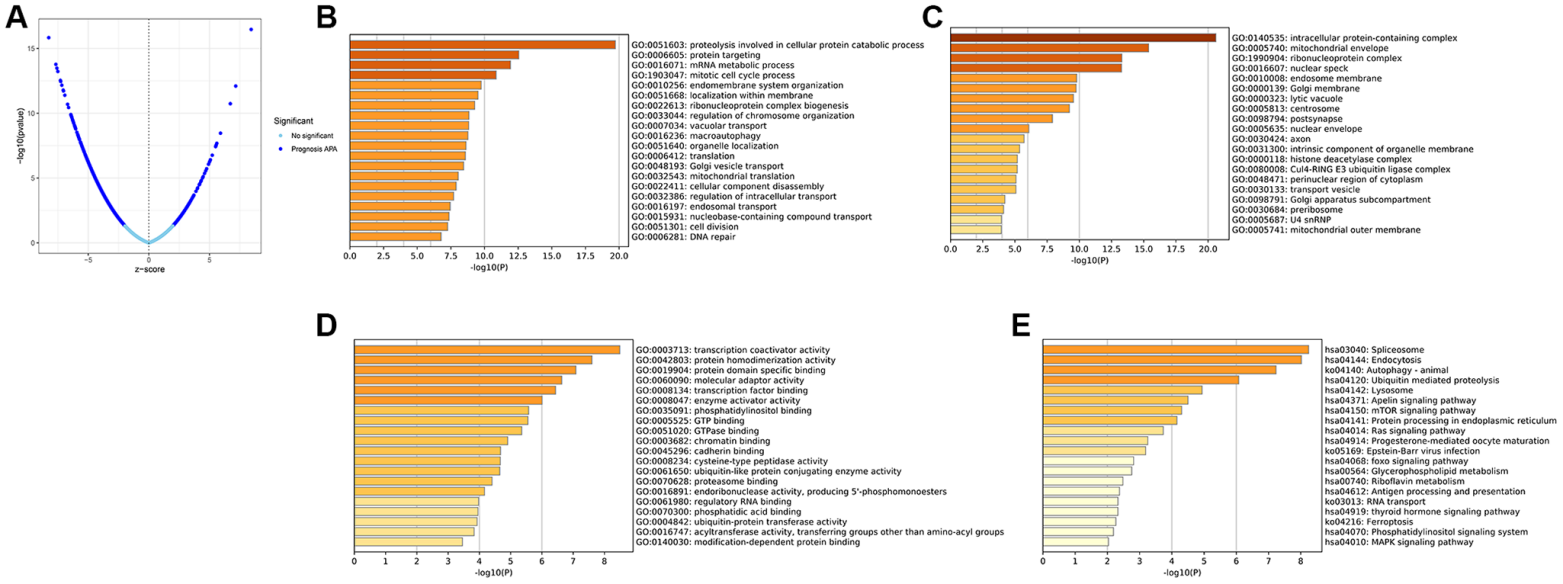
**Identification of the prognosis-related APA events and enrichment analysis of the corresponding genes of prognosis-related APA events.** (**A**) Volcano plot of prognosis-related APA events. The top 20 significant enrichment terms in BP (**B**), CC (**C**), and MF (**D**) in the GO analysis. (**E**) KEGG pathway analysis.

For further investigation of the impact of APA on prognosis, we randomized 511 LGG patients into either a training set (*n* = 256) or a test set (*n* = 255). After the analysis of all APA events in the training set by univariate Cox regression, a total of 1,213 prognostic-related APA events were screened out of 1,164 genes. These APA events were shown in the volcanic map (Figure 1A). Parental genes refer to genes involved in APA events, and their functions and pathways may reflect the potential role of APA events. To elucidate the potential biological functions of these parent genes related to the survival-related APA events in LGG, the GO enrichment and KEGG pathway analysis were performed on these genes. The GO and KEGG analyses highlighted that these genes were enriched in large quantities in RNA processing, protein synthesis, cell metabolism, and other processes, revealing the correlation between APA and these basic biological processes (Figure 1B–1E).

### Construction of prognostic APA signature

**Figure 2:**
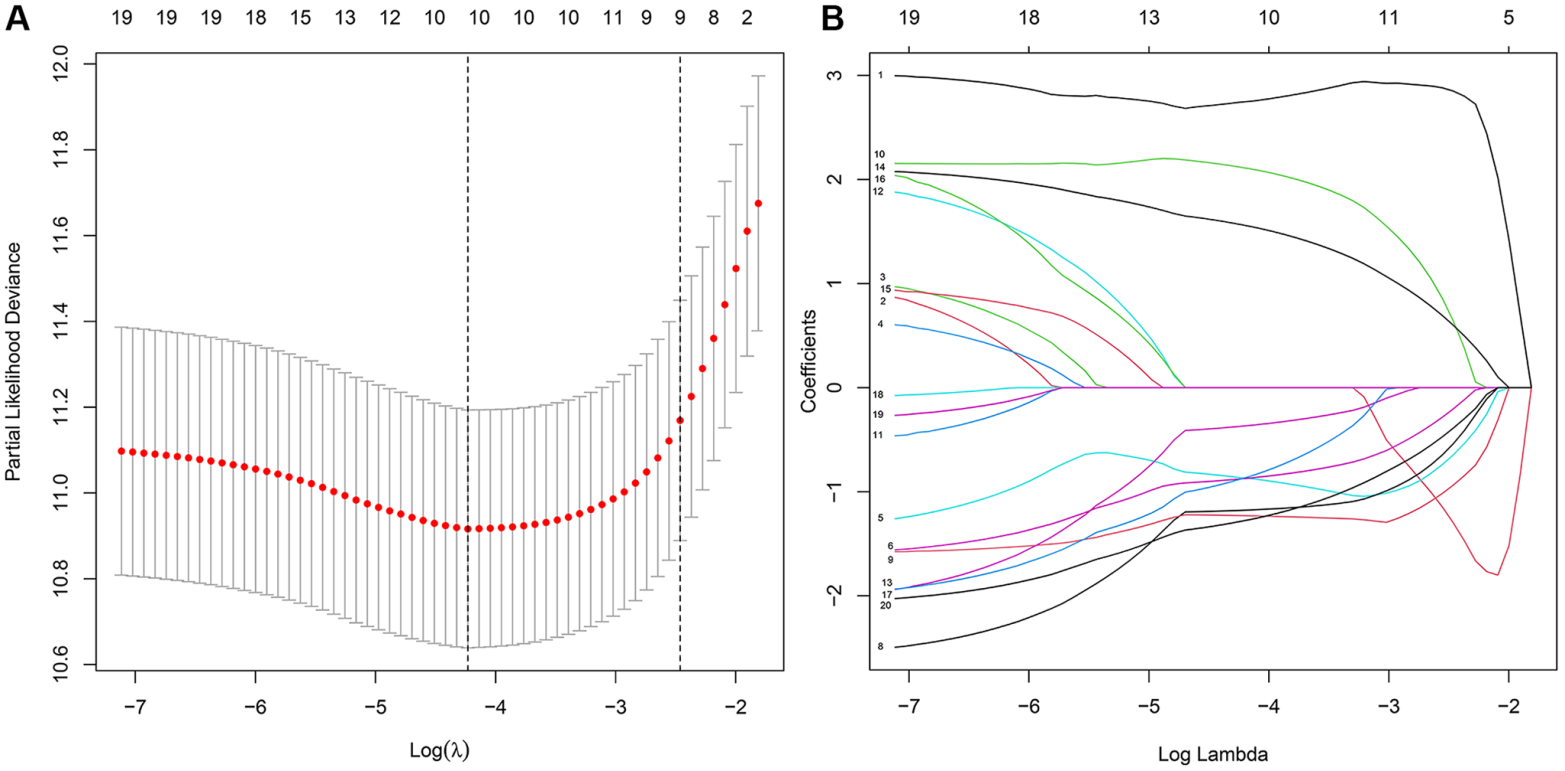
**The key features were identified using LASSO regression.** (**A**) Selection of the optimal parameter (lambda) via 5 times cross–validation. (**B**) LASSO coefficient profiles of the top 20 prognosis-related APA events.

**Figure 3:**
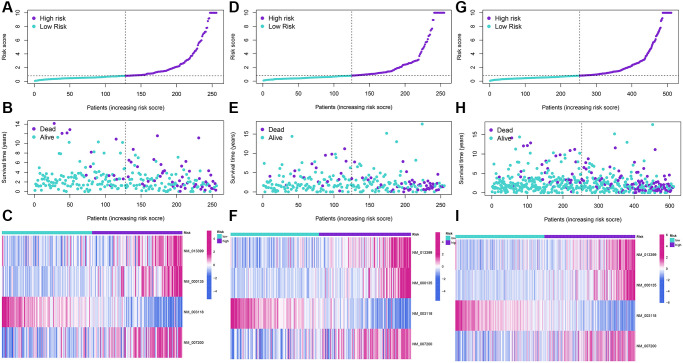
**Development and validation of a four-APA-based prognostic signature.** The risk score distribution, gene expression, and LGG patients’ survival status in the training (**A**–**C**), test (**D**–**F**), and whole sets (**G**–**I**) based on the signature.

**Figure 4:**
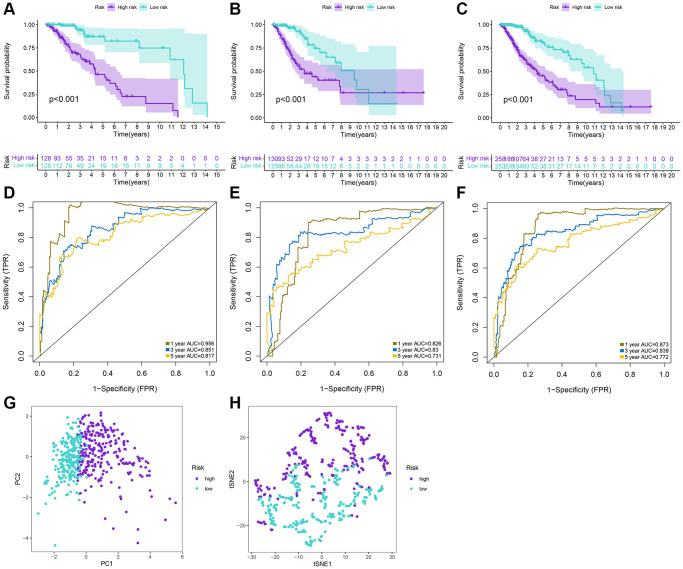
**Prediction performances of the signature for LGG patients.** (**A**–**C**) Kaplan-Meier analysis of high-risk and low-risk patients stratified by the median risk score in the training, test and whole sets. (**D**–**F**) Time-dependent ROC curves for 1-year, 3-year, and 5-year OS predictions by the signature in the training, test, and whole sets. (**G**, **H**) PCA and tSNE plots for LGG patients based on the risk groups.

**Figure 5:**
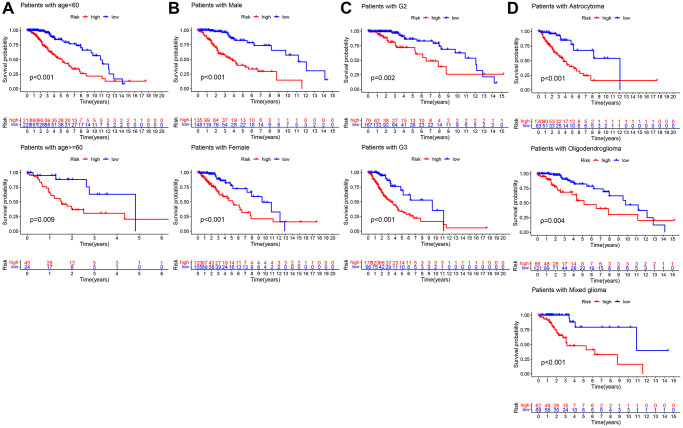
**Kaplan–Meier survival curves of LGG patients in different clinical subgroups.** (**A**) age. (**B**) Gender. (**C**) Grade. (**D**) Histology.

The LASSO regression analysis was performed for further screening of significant APA events from the top 20 most important prognostic APA events (Figure 2), which were considered as candidate genes for the construction of a prediction signature using the multivariate Cox regression analysis. In the end, a prognostic signature was constructed that consisted of four APA events. The coefficients of the four events were showed in Supplementary Table 1. The risk value for individual LGG patients was measured by following the formula, and all patients were classified into two groups called the high-risk and low-risk groups according to the median risk score (Figure 3A–3C). According to the Kaplan-Meier analysis these prognostic models successfully stratified patients with different results, it was observed that the patients that belonged to the high-risk group had a substantially shorter OS as compared to those in the low-risk group (Figure 4A). For the additional evaluation of the predictive power of the signature in 1-year, 3-year, and 5-year, the ROC analysis was conducted. The outcomes indicated that the signature had good prognostic performance in different years, with AUC values ranging from 0.817 to 0.956 (Figure 4D). Moreover, this study also verified the predictive power and accuracy of the signature in the training set and the whole set (Figure 3D–3I). The Kaplan-Meier survival analysis confirmed a substantial decline in the OS in high-risk groups of both datasets (Figure 4B, 4C). The AUC values of test set and whole set at 1-year, 3-year, and 5-year were 0.826, 0.830, 0.731 and 0.873, 0.839, 0.772, respectively (Figure 4E, 4F). According to the PCA and t-SNE analyses, patients in the two risk groups were distributed in two separate directions (Figure 4G, 4H). To confirm the general applicability of this signature, patients were subdivided by age, sex, grade, and pathological type. Considering the different stratified analysis results, the OS duration of LGG patients in the low-risk group was significantly longer in comparison to that in the high-risk group (Figure 5). As indicated by these findings, the signature can accurately and effectively predict the prognosis of patients with LGG.

### TMB and CNVs analysis

**Figure 6:**
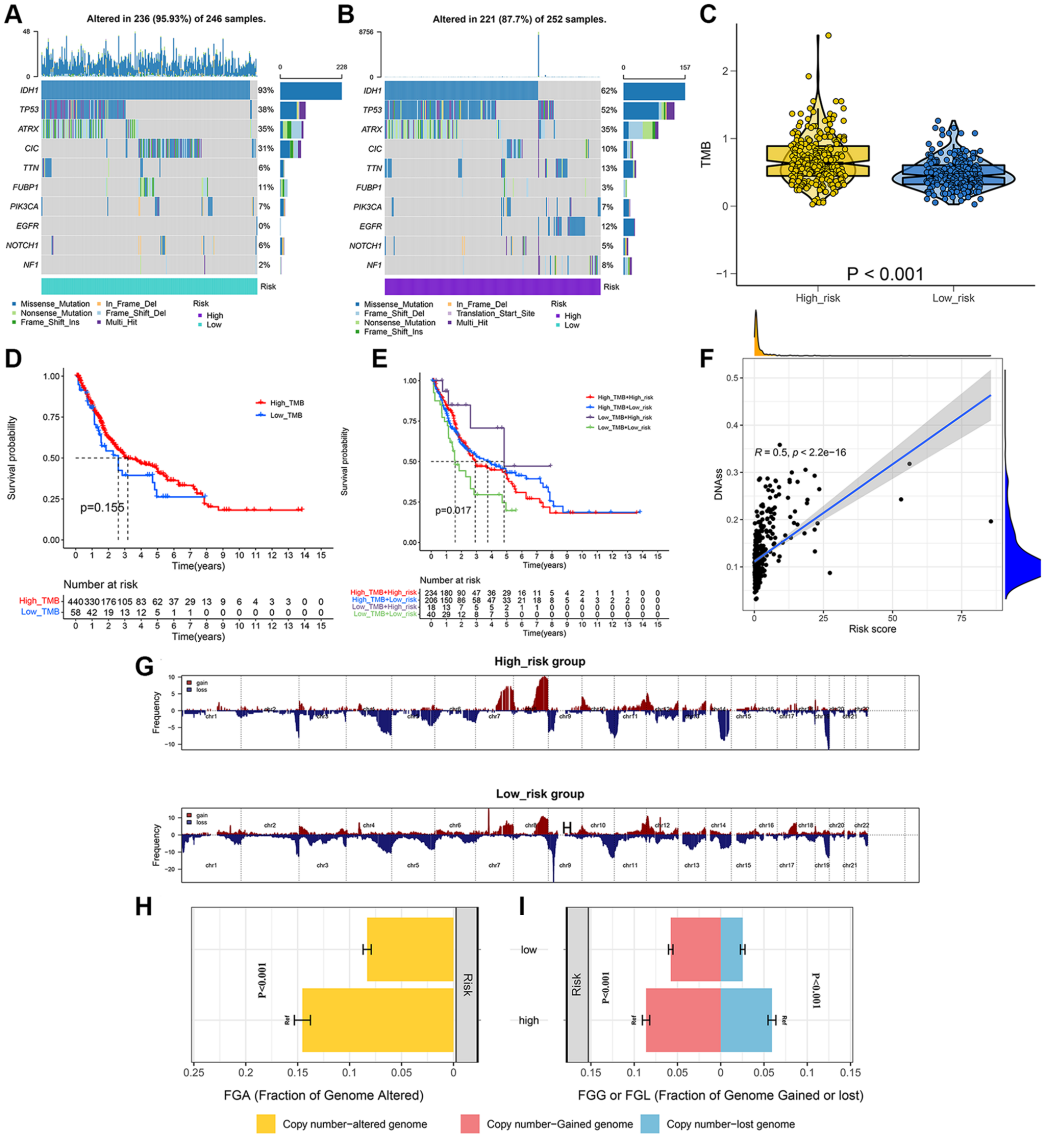
**Integrated comparisons of somatic mutation and CNVs between high-risk and low-risk groups in the whole set.** (**A**, **B**) Waterfall plots showing the mutation information of top 10 genes with the highest mutation frequency in two groups. (**C**) Distribution of TMB in two groups. (**D**) Survival curves for the OS of the high-TMB and low-TMB groups. (**E**) Survival curves for patients stratified by both TMB and signature. (**F**) Relationship between risk score and DNAss. (**G**) Gene fragments profiles with amplification (red) and deletion (blue) among the two groups. (**H**, **I**) Comparison of the fraction of the genome altered, lost, and gained between the two groups.

We analyzed and visualized somatic mutation data in LGG patients by distinguishing between the high-risk and low-risk groups. The top 10 drive genes with the highest variation frequency in the two risk groups were shown in Figure 6A, 6B. TMB of the two risk groups were calculated based on somatic mutation data, and the outcomes indicated that the TMB of the high-risk group was greatly enhanced as compared to that of the low-risk group (Figure 6C). We then identified whether TMB was an independent biomarker for LGG patients. LGG cohorts are split into high-TMB and low-TMB groups as per the median TMB value. According to the findings, the TMB cannot be used alone for predicting patient outcomes (Figure 6D). However, when the risk score and TMB were combined they could effectively predict LGG patient outcomes (Figure 6E). We also performed correlation analysis to explore the association of signature with tumor stem cells, and the results suggested that the risk score was negatively correlated with DNAss (Figure 6F). Additionally, we analyzed CNVs data using the GISTIC algorithm to find the gene regions with apparent amplification or deletion. The distribution of copy number changes in both the high and low-risk groups was demonstrated in Figure 6G. Overall, there were significant differences in the gene and frequency of copy number changes between the two groups. The frequency of gene changes, deletion, and gain was elevated in the high-risk group as compared to that in the low-risk group (Figure 6H, 6I).

### Analysis of immune function and immunotherapy

**Figure 7:**
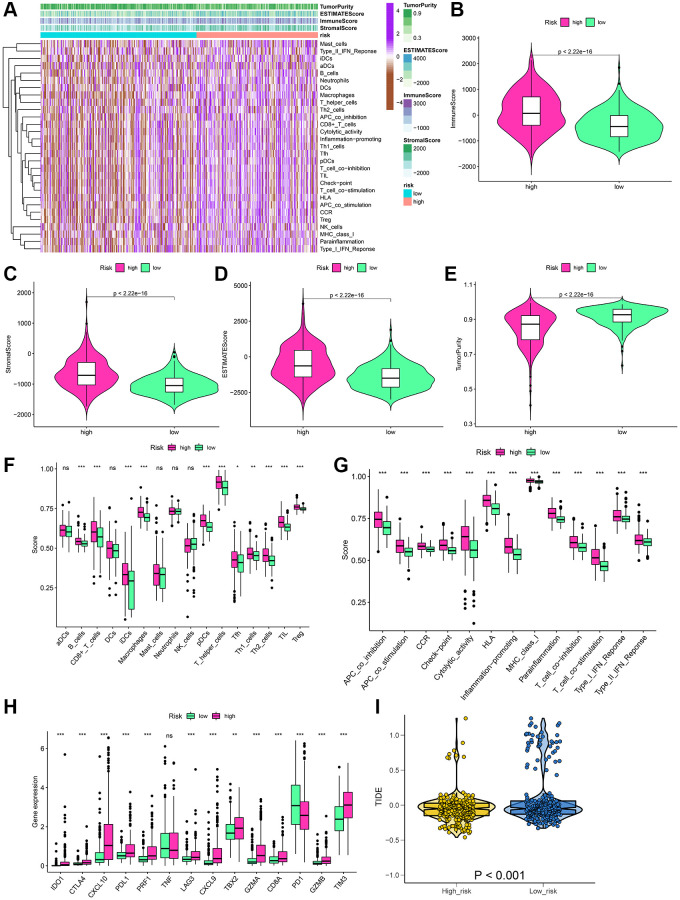
**Estimation of the immune status and response to immunotherapy based on the signature in the high-risk and low-risk groups for the whole set.** (**A**) Heatmap of the immune scores, stromal scores, tumor purity, ESTIMATE scores and immune-infiltrating cells in the two groups. (**B**–**E**) Violin plots for the immune scores, stromal scores, ESTIMATE scores, and tumor purity. (**F**–**H**) Boxplots of immune cells scores, immune-related functions scores and immune checkpoints expression. (**I**) Prediction of immunotherapy response according to TIDE score. ^*^P < 0.05; ^**^P < 0.01; ^***^P < 0.001; ns: no significance.

The materials and methods mentioned in the algorithm were used to assess the immune status of each LGG patient, as shown in the heatmap (Figure 7A). Moreover, the Wilcoxon test was performed for the comparison of the differences in individual cell markers among the high-risk and low-risk groups. The results indicated that stromal, immune, and ESTIMATE scores were substantially enhanced in the high-risk group as compared to those in the low-risk group, but tumor purity was lower in the low-risk group (Figure 7B–7E). Concerning the immune cells and functions, 11 types of immune cells and 13 immune function types were present in greater abundance in the high-risk group (Figure 7F, 7G). Additionally, 14 immune checkpoint genes between the two risk groups were compared. The outcomes indicated that 12 genes were highly expressed in the high-risk group (Figure 7H). We observed the TIDE scores to be closely correlated with immune checkpoint blocker response. As shown in Figure 7I, TIDE scores of the low-risk group patients were substantially enhanced as compared to those in the high-risk group, suggesting that LGG patients having high-risk scores showed more sensitivity to immune checkpoint blockade (ICB) treatment.

### Chemotherapeutic drug sensitivity

Antitumor drug therapy is the basic treatment for patients with LGG. The response of high and low-risk LGG patients to anticancer drugs was evaluated in this study by using the 10-fold cross-validation prediction signature through the GDSC database. Out of the 137 drugs, 29 drugs had IC50 values that differed among the two groups, suggesting that low-risk patients showed more sensitivity to these 29 drugs (Supplementary Figure 2). 16 drugs in particular, including Gefitinib, Lenalidomide, and Axitinib, had great potential for the treatment of LGG.

### Construction of prognostic nomogram

**Figure 8:**
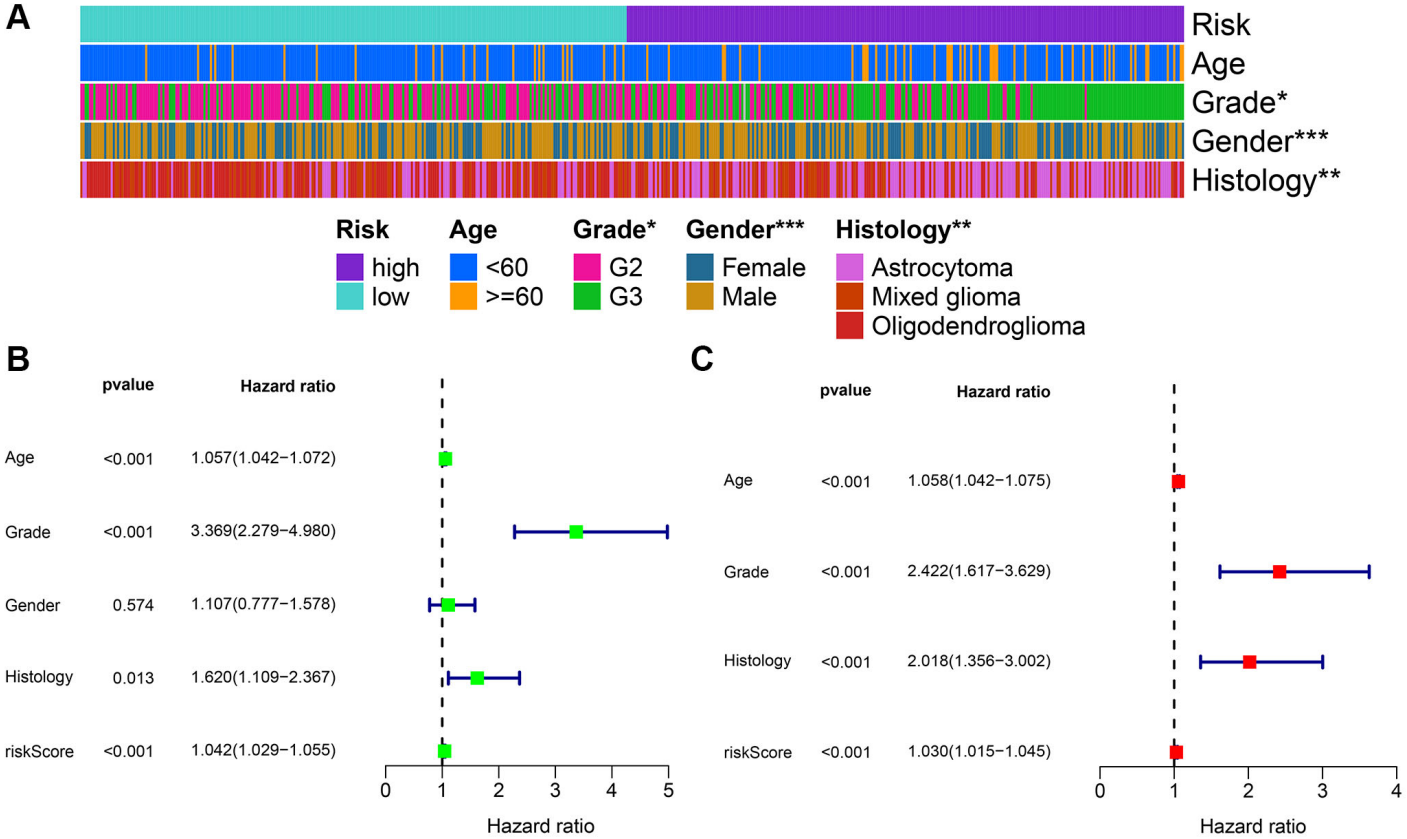
**Combined analysis of signature and clinicopathological characteristics in the whole set.** (**A**) Heatmap presents the distribution of clinical feature and corresponding risk score. (**B**) Univariate Cox regression analysis of clinical characteristics and signature. (**C**) Multivariate Cox regression analysis of clinical characteristics and signature. ^*^P < 0.05; ^**^P < 0.01; ^***^P < 0.001.

**Figure 9:**
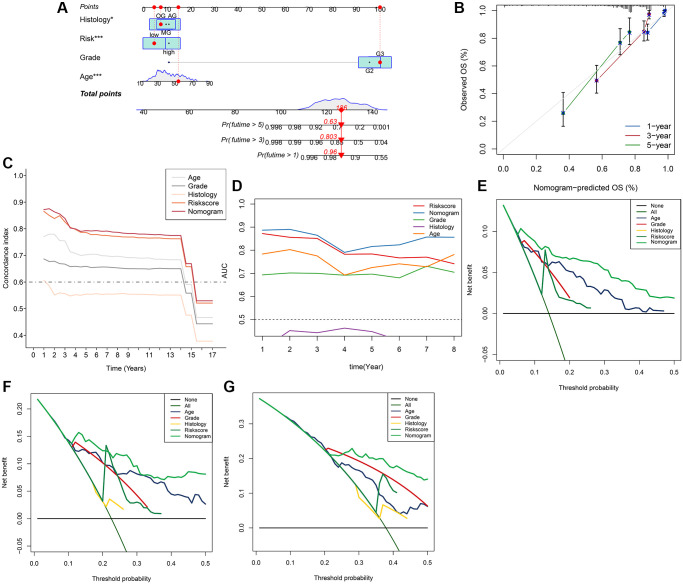
**Nomogram model for the prediction of LGG prognosis for 1-year, 3-year, 5-year OS in the whole set.** (**A**) Nomogram of applied to predict survival. (**B**) Calibration curve. (**C**) Graph showing concordance index changes over time. (**D**) AUC values of time-dependent ROC curves changes over time. (**E**–**G**) Decision curve analysis for 1-year, 3-year, 5-year OS.

Initially, the correlation between clinicopathological indicators and the signature was explored. The bar chart indicated prominent variations in gender, grade, and pathological type among the high and low-risk groups (Figure 8A). Subsequently, a nanogram was created on the basis of outcomes of univariate and multivariate Cox regression (Figure 8B, 8C), including pathological type, sex, age, and risk score (Figure 9A). The OS was calculated for all patients and was predicted at 1-year, 3-year, and 5-year. It can be seen from the calibration curve that the results predicted by the nomogram supported the actual outcomes (Figure 9B). The results of the time ROC curve and C index curve showed that AUC and C index at different times were the largest in the nomogram (Figure 9C, 9D). Furthermore, as compared with the risk score signature, the nomogram had improved the net benefit of clinical patients even more (Figure 9E–9G).

### Potential regulatory network of APA

**Figure 10:**
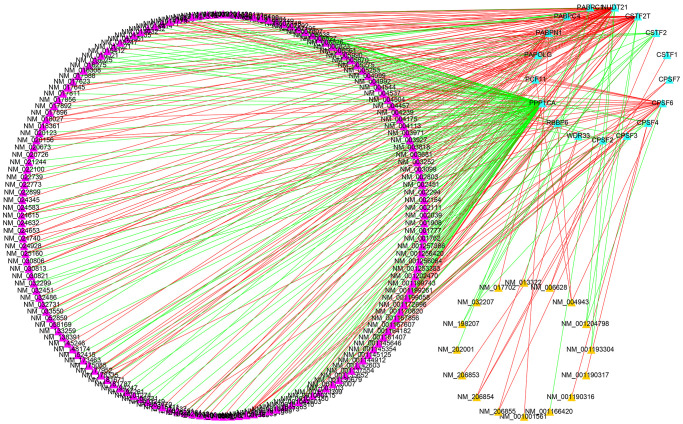
**Construction of a survival-associated CRs-APAs network.** Blue triangles, purple triangles and yellow triangles were CRs, good prognosis events and poor prognosis, respectively. Red/green lines represent positive/negative correlations between nodes.

For a detailed analysis of the potential regulatory mechanism of survival-related APA events in LGG cohorts, an interaction network of APA events and key regulators were designed. The Spearman test was used to observe the correlation between PDUI values and CRs gene expression levels of OS-related APA events. Significant relationship pairs with the correlation coefficient >0.5 and *P*-value <0.001 were selected to create the correlation network. As demonstrated in Figure 10, the expression levels of 17 CRs (shown as the blue triangles) were greatly correlated with 180 survival-related APA events, including the 164 APA events with good prognosis (shown as the purple triangles) and 16 APA events with poor prognosis (shown as the yellow triangles). Interestingly, it was found that the ratios of SFs increased (shown as the red lines) and decreased (shown as the green lines) were 1:1 for both APA events with poor survival prognosis and the APA events with good survival prognosis.

## DISCUSSION

Heterogeneity in patients is a key contributing factor in the poor prognosis of glioma, and multifaceted evaluation can help in improving the precision of prognosis prediction for such patients [18]. Therefore, the latest version of gliomas’ classification by the WHO incorporates molecular features into the classification criteria, thereby improving the homogeneity of clinical outcomes in patients with the same subtype [19]. Nevertheless, as one of the histological subtypes of glioma, patients with LGG vary greatly in survival and lack effective prognostic markers. APA is a common post-transcriptional regulatory mechanism in eukaryotic organisms. The differential expression of APA can be caused by affecting the stability of transcripts, their output to the cytoplasm, and their translation efficiency [5]. Studies have found that extensive APA occurs in the pathophysiological process of numerous diseases, including cancer. In such diseases, APA events are emerging as potential biomarkers that can be used in clinical practice. The majority of the dysfunctional APA events result in the production of transcript isomers with variable lengths of 3′UTRs. These are often associated with various clinical traits. These APA events do not depend on the commonly used molecular data, such as gene expression and somatic mutations [20] and they are associated with disease prognosis, recurrence, tumor subtypes, and staging of multiple cancers [21–24]. However, there are few systematic studies on the function of APA events in LGG.

In the current research, systematic identification and analysis of survival-related APA events was performed in LGG patients from the TCGA database and 1,213 survival-related APA events were discovered in 1,164 genes. By performing the GO and KEGG analysis of parental genes of these prognostic APA events, it was observed that these events were largely involved in RNA processing, protein synthesis, cell metabolism, and other processes, such as proteolysis involved in cellular protein catabolic process, mRNA metabolic process, and endocytosis. Meanwhile, we constructed a correlation network of CRs-APA to reveal the potential regulatory mechanism of prognostic APA events. For identifying the prognostic importance of APA events, a prognostic prediction signature was constructed for LGG based on the screening of the top 20 survival-related APA events. Patients were sorted into two groups called the low-risk and high-risk groups on the basis of the median risk score. The Kaplan-Meier method indicated that the OS of the training set, test set, and whole set in the high-risk group was worse as compared to that of the low-risk group. This observation was consistent with the ROC curve analysis. Nomogram contribute to visualization of statistical models, calculation of predicted values and graphical evaluation of important indicators [25]. It has been widely used to predict prognostic risk and therapeutic effect of patients. Here, a nomogram scoring system was established by the combination of the signature and independent prognostic indicators. The signature has better predictive power than other independent prognostic indicators (Figure 9C, 9D). More importantly, nomogram presented the most quantitative prognostic prediction power and net benefit factor for clinical purposes (Figure 9C–9G). This demonstrates the superiority of combining multiple indicators to predict patient outcomes. All the parent genes of APA in the signature were previously confirmed to be related to tumors by external studies. The CDIP1 has been considered as a target gene of P53, which is upregulated in response to DNA damage and is a key downstream effector of p53-dependent apoptosis. CDIP1 induces apoptosis by providing a link between internal apoptosis mediated by P53 and external apoptosis mediated by death receptors [26]. Not surprisingly, CDIP1 also plays an important role in tumor cell apoptosis. Zhou et al. found that IL-33 plays a carcinogenic role by inhibiting the expression of CDIP1, thereby reducing apoptosis of non-small cell lung cancer [27]. The most widely mutated gene in fanconi anemia (FA) known as FANCA is a member of the FA core complex that recognizes interchain cross-linking and induces subsequent DNA repair [28, 29]. Previous studies have highlighted that FANCA gene mutations are closely related to the occurrence and development of different types of tumors. For instance, mutations in the FANCA gene can increase cellular activities including transcriptional basal efficiency or transcriptional regulation, increasing breast cancer risk [30]. The loss of FANCA function in the germline is considered to be a pathogenic mutation in the development of prostate cancer. FANCA-associated DNA repair mutations occur more frequently in prostate cancer with high Gleason grade as compared to the low Gleason grade, and the prognosis is generally worse [31]. SPARC is a major gene affecting cellular interactions, extracellular matrix remodeling, and bone mineralization [32]. It is usually expressed by mesenchymal cells and can inhibit or promote cancer in different tumor types. Some researchers have discovered that SPARC is highly expressed in pancreatic cancer (PC) tissues, and overexpression of SPARC in PC cells can induce epithelial mesenchymal transition (EMT) and stimulate the migration and invasion of cancer cells [33]. In endometrial cancer (EC), low SPARC expression is related to aggressive EC phenotype and poor prognosis. In addition, down-regulation of SPARC promotes the EMT process and enhances EC cell proliferation and invasion [34]. The AKAP13 is an anchor protein found in the Rho signaling pathway. AKAP13 is expressed increasingly in hepatocellular carcinoma, but it is not expressed in healthy adult liver. Its overexpression in hepatocellular carcinoma cell lines promotes cell proliferation and leads to increased levels of downstream ERK and cyclin D1 [35]. AKAP13 plays a major role in PKA-induced phosphorylation of ER, which is a significant cause of tamoxifen resistance in breast cancer cells and cancer patients [36].

Recently, many studies have stressed the significance of immunotherapy in glioma and it has become a research hotspot [37]. Due to the heterogeneity of gliomas, the therapeutic effect of gliomas is not entirely satisfactory [38]. TMB is a potential biomarker for predicting immunotherapy outcomes for different types of cancers [39, 40]. At the same time, CNVs may provide a better predictor of immunotherapy response than traditional biomarkers. These two molecular characteristics provide new perspectives and good practical methods for immunotherapy [41]. Consequently, we explored variation in genetic mutations between the high-risk and low-risk groups. The high-risk group had substantially increased percentages of TMB and CNV as compared to the low-risk group. The immune infiltration level greatly affects the prognosis of tumor patients. The tumor immune microenvironment contains stroma and immune cells and is associated with immunotherapeutic responses. Additionally, targeting tumor immune checkpoints can serve as a new technique for killing tumor cells, and the expression of immune checkpoints is related to immunotherapeutic responses [42, 43]. Considering the importance of immune status in immunotherapy, the relationship between the signature and immune cell infiltration and immune checkpoint was identified in this study. The ESTIMATE and ssGSEA algorithms suggested that the high-risk group had a higher proportion of immune and stromal cells, stronger immune function, and lower tumor purity. Immune checkpoint expression analysis yielded similar results for immune cell infiltration, again with higher levels in the high-risk group. Due to the lack of open data on LGG patients receiving immunotherapy and APA testing, the TIDE algorithm was used for preliminary identification of the response of this cohort to immunotherapy. The outcomes showed that the high-risk group had a lower TIDE score and might have increased sensitivity to immunotherapy. Chemotherapy and targeted gene therapy have been shown to have many advantages in prolonging survival in LGG patients [44, 45]. Hence, it is important to predict the treatment response of LGG patients to chemotherapy agents and molecularly targeted antitumor agents. The data in this research indicated that patients in the low-risk groups were more sensitive to 16 drugs, including Gefitinib, Lenalidomide, and Axitinib, as compared to those in the high-risk groups. These findings showed that the APA signature had a close correlation with immunotherapy and chemotherapy and it can be used to select individualized treatment strategies for LGG patients.

There are some deficiencies in this study that can be highlighted. For instance, this research was based on one study cohort only. Only internal validation was performed in the absence of another dataset for external validation. Moreover, this signature has not been clinically validated with a large sample, so its clinical practicality cannot be evaluated directly. Lastly, due to the lack of *in vitro* or *in vivo* experiments, the specific molecular mechanisms of these biomarkers remain unclear. In future studies, we will conduct detailed studies for the validation of our current results.

In conclusion, this study comprehensively analyzed the specific role of APA events in LGG and identified survival-related APA events for the first time. Furthermore, we created prediction signatures based on these events that can accurately stratify the risk and predict the prognosis of LGG patients. In addition, APA events in the signature are expected to be targets for LGG treatment in the future.

## Supplementary Materials

Supplementary Figures

Supplementary Table 1
