# Supplementary material for: A new signature based on alternative polyadenylation for prognostic prediction and therapeutic responses in low-grade glioma
Source: Aging (Albany NY). 2022 Jan 18;14(2):826–44. doi: 10.18632/aging.203844 (PMC8833112; doi:10.18632/aging.203844)
Supplement: Supplementary Figures [file aging-14-203844-s001.pdf]

SUPPLEMENTARY FIGURES

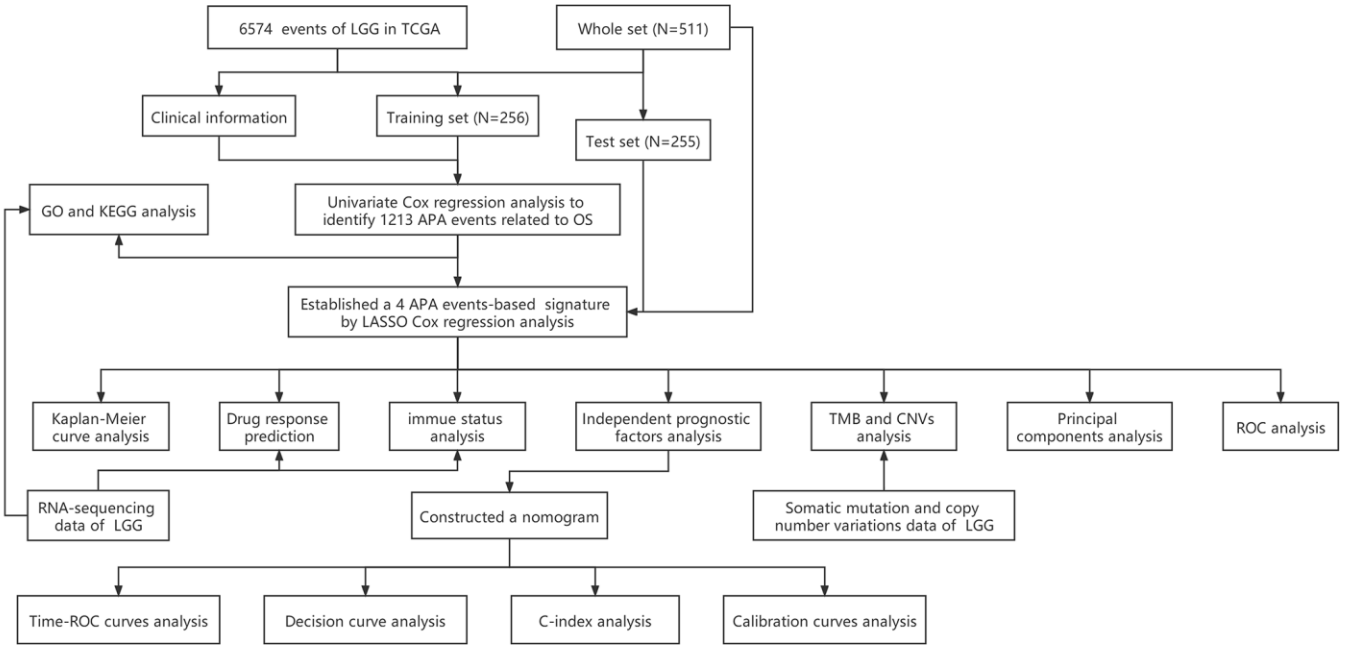

Supplementary Figure 1. Flow chart of the present study.

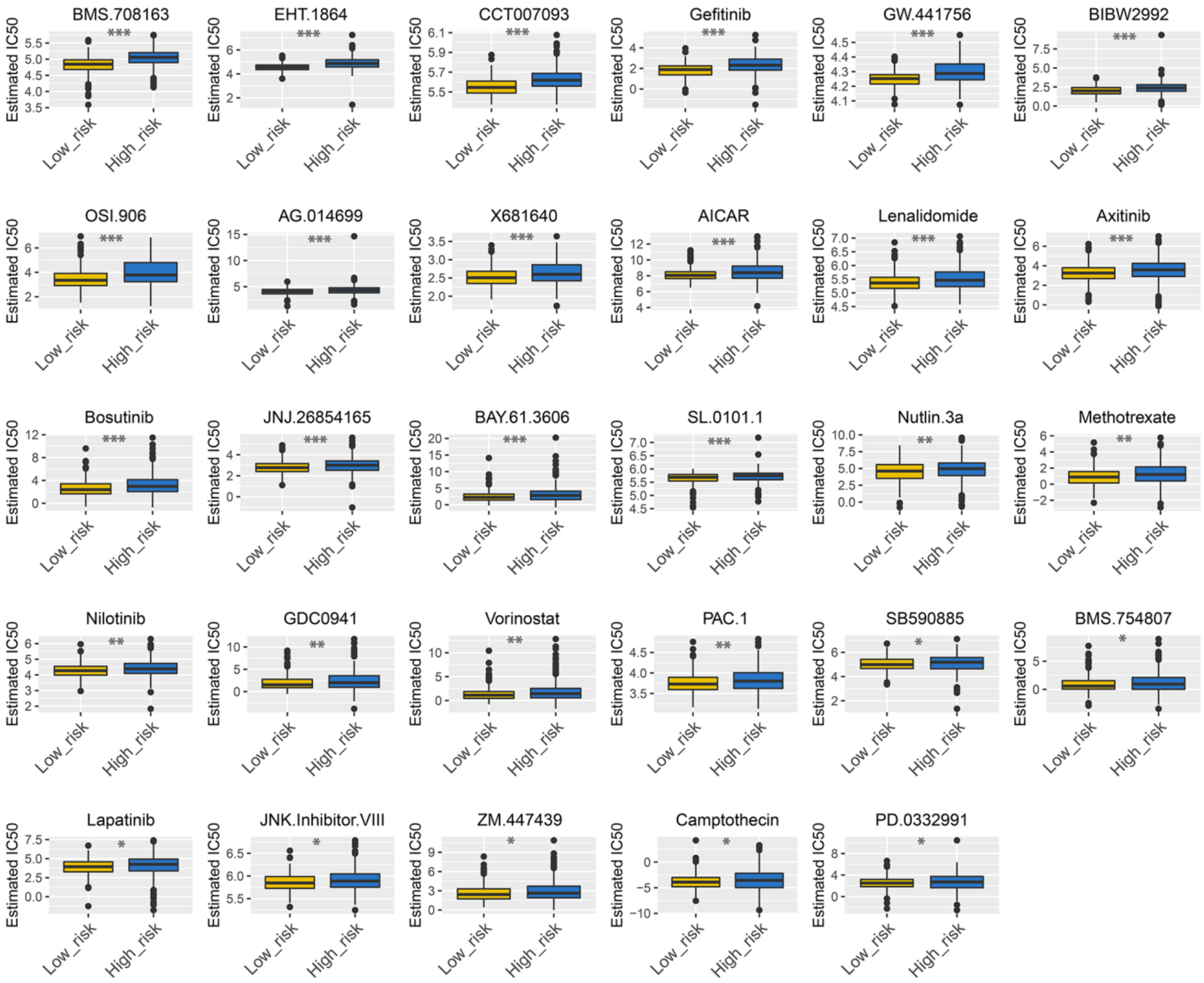

Supplementary Figure 2. Boxplots of estimated IC50 values of potential compounds between high-risk and low-risk groups. $^{*}P < 0.05$; $^{**}P < 0.01$; $^{***}P < 0.001$.
