# Supplementary material for: A new signature based on alternative polyadenylation for prognostic prediction and therapeutic responses in low-grade glioma
Source: Aging (Albany NY). 2022 Jan 18;14(2):826–44. doi: 10.18632/aging.203844 (PMC8833112; doi:10.18632/aging.203844)
Supplement: Supplementary Table 1 [file aging-14-203844-s002.pdf]

SUPPLEMENTARY TABLE

Supplementary Table 1. The coefficients of the four events.

Id	Coef	HR	HR.95L	HR.95H	*p* value
\`NM_013399|C16orf5|chr16|−\`	5.846687	346.0859	4.230633	28311.47	0.009273
\`NM_000135|FANCA|chr16|−\`	7.129974	1248.845	11.95803	130423.9	0.002645
\`NM_003118|SPARC|chr5|−\`	−4.96019	0.007012	0.000385	0.127679	0.000808
\`NM_007200|AKAP13|chr15|+\`	1.243714	3.468473	0.671534	17.91467	0.13764
